# Capturing Genetic Diversity and Selection Signatures of the Endangered Kosovar Balusha Sheep Breed

**DOI:** 10.3390/genes13050866

**Published:** 2022-05-12

**Authors:** Olusegun O. Adeniyi, Rebecca Simon, Hysen Bytyqi, Waltraud Kugler, Hajrip Mehmeti, Kaltrina Berisha, Mojca Simčič, Mohamed Magdy, Gesine Lühken

**Affiliations:** 1 Institute of Animal Breeding and Genetics, Justus Liebig University, 35390 Giessen, Germany; olusegun.o.adeniyi@agrar.uni-giessen.de (O.O.A.); rebecca.simon@agrar.uni-giessen.de (R.S.); mohamed.ismail-magdy-saadeldin-sabri@agrar.uni-giessen.de (M.M.); 2 Faculty of Agriculture and Veterinary, University of Pristina, 10000 Pristina, Kosovo; hysen.bytyqi@uni-pr.edu (H.B.); hajrip.mehmeti@uni-pr.edu (H.M.); kaltrina.berisha@uni-pr.edu (K.B.); 3 SAVE Foundation, 9000 St. Gallen, Switzerland; waltraud.kugler@save-foundation.net; 4 Department of Animal Science, Biotechnical Faculty, University of Ljubljana, SI-1000 Ljubljana, Slovenia; mojca.simcic@bf.uni-lj.si

**Keywords:** *ovis aries*, conservation/preservation, population structure, ROH, GO TERMS, Pramenka, Balkan

## Abstract

There is a growing concern about the loss of animal genetic resources. The aim of this study was to analyze the genetic diversity and potential peculiarity of the endangered Kosovar sheep breed Balusha. For this purpose, a dataset consisting of medium-density SNP chip genotypes (39,879 SNPs) from 45 Balusha sheep was generated and compared with SNP chip genotypes from 29 individuals of a second Kosovar breed, Bardhoka. Publicly available SNP genotypes from 39 individuals of the relatively closely located sheep breeds Istrian Pramenka and Ruda were additionally included in the analyses. Analysis of heterozygosity, allelic richness and effective population size was used to assess the genetic diversity. Inbreeding was evaluated using two different methods (*F_IS_*, *F_ROH_*). The standardized *F_ST_* (*d_i_*) and cross-population extended haplotype homozygosity (XPEHH) methods were used to detect signatures of selection. We observed the lowest heterozygosity (*H_O_* = 0.351) and effective population size (Ne_5_ = 25, Ne_50_ = 228) for the Balusha breed. The mean allelic richness levels (1.780–1.876) across all analyzed breeds were similar and also comparable with those in worldwide breeds. *F_ROH_* estimates (0.023–0.077) were highest for the Balusha population, although evidence of decreased inbreeding was observed in *F_IS_* results for the Balusha breed. Two Gene Ontology (GO) TERMs were strongly enriched for Balusha, and involved genes belonging to the melanogenesis and T cell receptor signaling pathways, respectively. This could result from selection for the special coat color pattern of Balusha (black head) and resistance to certain infectious diseases. The analyzed diversity parameters highlight the urgency to preserve the local Kosovar Balusha sheep as it is clearly distinguished from other sheep of Southeastern Europe, has the lowest diversity level and may harbor valuable genetic variants, e.g., for resistance to infectious diseases.

## 1. Introduction

**Figure 1:**
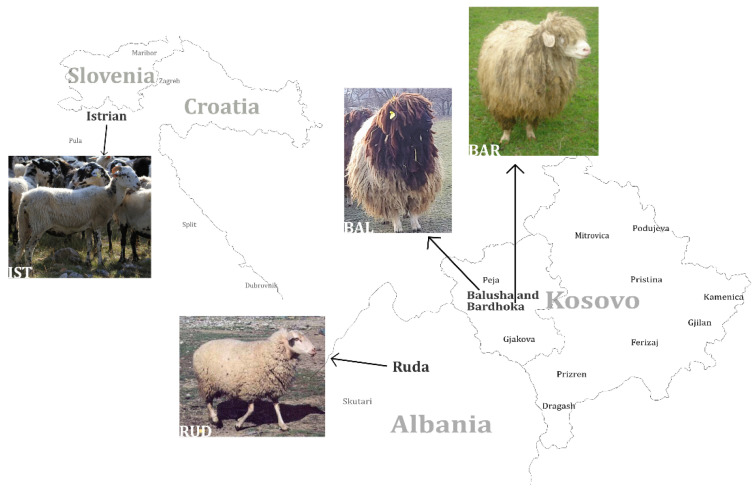
Geographic distribution of four analyzed sheep breeds in southeastern Europe. The black-headed Balusha (BAL), the white Bardhoka (BAR), originating from the same area in western Kosovo, the Albanian Ruda (RUD; photo: A. Hoda) and the Istrian Pramenka (IST; photo: V. Rezar), the latter one sampled in Slovenia. Please note that the proportions and distances of the map are not displayed realistically, they are only for orientation.

Since the domestication of sheep at around 10.000 BC [1], many breeds with high diversity in appearance, reproductive traits and performance characteristics have developed worldwide through breeding and selection. The Balkans are considered to be the main entry point in the immigration of agriculture in general, and sheep especially, to present-day Europe during the Neolithic period [2,3,4,5]. There is evidence that in the 20th century, crossbreeding of Merinoland sheep with some of the local Pramenka breeds took place in the Balkans [6]. However, due to changes in agriculture and the loss of economic importance of sheep in Europe [7], numbers of flocks are decreasing and many traditional breeds that are not used in commercial husbandry are threatened with extinction [8] (breed examples [9]). This is also the case in Kosovo. Four Pramenka breeds (rough-wooled types), which are mainly used for triple purposes, milk–meat–wool [10], are considered to be local in Kosovo: Sharri, Kosova, Bardhoka (BAR, also known as White Metohian Pramenka) and Balusha (BAL). BAR and BAL sheep (Figure 1) are kept in areas in the southwestern part of the country (Dukagjini plane), where they were mainly selected for traits associated with milk production [11].

The medium-size Balusha is coarse-wooled with a black head and neck while the fleece and legs are white. Only rams are horned with screw-shaped horns, whereas ewes are polled. In contrast, the medium-sized coarse-wooled Bardhoka is completely white. The majority of rams have spiral horns but some are polled, which is considered untypical. Both breeds are adapted for milk production based on grazing of natural pastures [11,12]. Medium-sized RUD is a breed with clear Merino introgression, which was already confirmed by genetic analysis [2]. RUD is used for triple purposes, with white medium-quality fleece, legs and head, but rarely, individuals can be completely black. The rams carry big spiral horns, while females are polled [12,13]. The IST is widespread in the western region of the Balkan Peninsula. The large IST, which is used for milk production, has a white coarse fleece with a different amount of black spots, to the extent that some animals are nearly black. The rams are always horned, while ewes can be both polled or horned. IST are known for the high-fat content in their milk [6,12].

Nowadays, especially the black-headed BAL sheep have become rare. It is assumed that 70% of the whole population, which consists of only 300 individuals [14], is kept by only two breeders. In addition, there is the possibility of unplanned cross-breeding of BAL with BAR, since both breeds are reared together in the same area (Dukagjini region) under an extensive system of farming [11]. Therefore, the breed is at a very high risk of extinction and loss of diversity. Preserving original breeds also means preserving characteristics and traits that may become important with changing requirements, e.g., by the farmers or environmental conditions, offering future breeding options [8,15,16]. The importance of regional breeds as a cultural heritage, which should be preserved, is also a factor in favor of preserving old breeds e.g., [7,17,18].

The Illumina Ovine 50K SNP chip [19] is a powerful tool to analyze the genetic diversity within a breed and across breeds and to determine the status quo of populations. This was confirmed in many studies on sheep diversity e.g., [20,21,22,23,24]. Various approaches are available to determine the degree of inbreeding and thus to assess the diversity of a breed as a whole and of individual animals. Classically, the expected and observed heterozygosity, the effective population size (Ne) and the Wright’s F statistical indices (Fis) can be used [25]. Moreover, with genome-wide SNP data, it is possible to quantify the extent of inbreeding, determined by the runs of homozygosity (ROH) [26], even with missing pedigree information [27]. The different lengths of the segments containing homozygous loci can be used to define whether the inbreeding occurred recently or can be rated as an ancient event [28].

Regions with high ROH are regions that also carry signatures of selection [29]. Different methods are available to detect those signatures of selection [30,31] that indicate patterns of reduced diversity close to genes that have been under strong selection, artificially or naturally caused, in a certain population [32]. The *d_i_* statistics is an established method used to calculate the locus-specific divergence of a specific breed against other breeds [30]. The cross-population extended haplotype homozygosity (XPEHH) is a haplotype-related analysis that detects differences between two populations in which a selected allele is fixed or approaching fixation in one population while still polymorphic in the other population [31].

The diversity of some sheep breeds from Eastern Europe was studied and evaluated, mainly by using microsatellite markers, but the BAL breed has not yet been considered [2,33,34]. With our study, we aimed to determine the genetic diversity and population structure of the Kosovar sheep breed BAL for the first time. Special attention is paid to the genetic differentiation of this breed from the BAR breed kept in the same region. Additionally, published Ovine 50K SNP chip data from the two relatively geographically close sheep breeds Istrian Pramenka (IST) and Ruda (RUD) (Figure 1) were included for comparison. ROH were analyzed and signatures of selection based on *d_i_* statistics as well as on XPEHH were compared. The results should provide a valid basis for the justification and establishment of conservation programs for the endangered BAL breed in a further step.

## 2. Materials and Methods

### 2.1. Animal Samples and Data

For this study, blood samples of BAL (*n* = 45) and BAR (*n* = 29) sheep breeds were collected from two farms in Kosovo (Figure 1). All animals were females, except for a single male in the BAL sample set. Unrelated animals were sampled where possible to avoid genetic relation. DNA was extracted due to manufacturer’s instructions from the blood samples using the NucleoSpin Blood Kit (Macherey-Nagel, Düren, Germany) and genotyped with the Ovine 50K SNP BeadChip (Illumina, San Diego, CA, USA). In addition, published Illumina Ovine 50K SNP BeadChip data from the sheep breeds Istrian (IST, *n* = 23) and Ruda (RUD, *n* = 16) were available from a recent study on Balkan sheep breeds (Figure 1) [2]. These data were merged and a total of 39,879 autosomal SNPs were available for the analysis. Data were filtered to exclude loci with minor allele frequency (<5%), SNPs with a low call rate (<95%) and animals with more than 5% of missing genotypes. To overcome the effect of closely related animals, the data were checked for relatedness which was estimated as a proportion of identity-by-descent (IBD) between animal pairs using *--related* option in KING v2.2.4 [35] and one of each animal pair with an IBD proportion > 0.35 was removed. After quality control (QC) procedures using PLINK v1.90 [36], a total of 39,214 autosomal SNPs and 92 samples (Table S1) remained in the dataset for genomic-based diversity analysis.

### 2.2. Analysis of Population Structure and Genetic Diversity

The above dataset was linkage disequilibrium (LD)-pruned using the *--indep-pairwise* function in PLINK v1.90 (this version of PLINK was also used for other analyses with that program; © 2022 Shaun Purcell, GNU General Public License). One of a pair of SNPs in high LD (r^2^  >  0.5) within a window size of 50 SNPs and with a window slide of 5 SNPs was removed. Pairwise identity-by-state (IBS) distances and principal component analysis (PCA)-based multidimensional scaling analysis were performed in PLINK, and a plot showing the first two principal components was constructed with the package “ggplot2” [37] in R [38]. The observed heterozygosity (*H_O_*) and expected heterozygosity (*H_E_*) were calculated using HIERFSTAT R package [39]. Additionally, rarified allelic richness and private allelic richness were estimated with the ADZE v1.0 software [40] using a standardized sample size for each breed. The Wright inbreeding coefficient was estimated as $F_{IS}=\frac{H_{E}-H_{O}}{H_{E}}$, where *H_O_* and *H_E_* are observed and expected heterozygosities, respectively [41]. Effective population size (Ne) for all breeds was calculated based on LD using the SNeP package [42]. Default parameters were adjusted for sample size, recombination rate according to [43], and occurrence of mutation as suggested by [44]. The Ne five (Ne_5_) and fifty (Ne_50_) generations ago were estimated. The ADMIXTURE program (Version 1.3.0; [45] was employed to determine the ancestry and results were visualized using “pophelper” R package version 2.3.0 [46]. Clustering was calculated for K values from 2 to 4 and optimal number of clusters was determined by adding the --cv flag [45].

### 2.3. Estimation of Runs of Homozygosity

Runs of homozygosity (ROH) were estimated using the QC data and *--homozyg* function in PLINK. One missing SNP, one heterozygous SNP, and ROH longer than 1Mb were allowed per run. A scanning window threshold of 0.05, a sliding window of 50 SNPs, a maximum gap between consecutive SNPs set to 250 kb, and a minimum average SNP density of more than one SNP/100 kb were used. The minimum number of SNPs (l) per run was calculated as suggested by [43]. $l=\frac{\log_{e}\frac{\alpha }{n_{{\mathrm{sn}}_{i}}}}{\log_{e}\left(1-\mathrm{het}\right)},$ where α is the percentage of false-positive ROH (set to 0.05 in this study), ns is the number of SNPs per individual, n_i_ is the number of individuals, het is the mean heterozygosity across all SNPs. Calculated l was 39 for BAL, 36 for BAR and 34 for IST and RUD. The total and mean ROH length were estimated for each individual and breed. In addition, the total and mean ROH numbers for each individual and breed were determined. To compare ROH frequency between breeds, ROH segments were categorized into 3 length classes (1–5, >5–10 and >10 Mb).

The inbreeding coefficient of ROH (*F_ROH_*) was determined according to [47]: $F_{ROH}=\sum \frac{L_{\mathrm{ROH}}}{L_{\mathrm{AUTO}}}$, where L_ROH_ is the sum of the length of all ROH per animal and L_AUTO_ is the total autosomal SNP coverage (2.648 Gb). The average *F_ROH_* per breed was determined for L_ROH_ above 1, 5 and 10 Mb, which is indicative of inbreeding up to 50, 10 and 5 generations ago, respectively [48,49,50]. Furthermore, box plots were constructed to show the within-breed distribution of *F_ROH_* per breed for L_ROH_ above 1 Mb.

### 2.4. Detection of Signatures of Selection

To determine selection signatures of BAL, the *F_ST_* and the cross-population extended haplotype-based homozygosity score test (XPEHH) [31] was employed using the QC data. Pairwise *F_ST_* [51] values of each SNP were estimated for BAL against a combination of all other sheep breeds in this study using the VCFtools v0.1.15 software. After this, the *F_ST_* values obtained were standardized (*d_i_*) according to the method published by [30]: $d_{i}=\sum_{j\neq i}\frac{F_{ST}^{ij}-E\left[F_{ST}^{ij}\right]}{sd\left[F_{ST}^{ij}\right]}$ where $E\left[F_{ST}^{ij}\right]$ and $sd\left[F_{ST}^{ij}\right]$ denote the expected value and standard deviation of *F_ST_* between breeds *i* and *j* calculated from all 39,214 SNPs. The locus-specific divergence calculated using the *d_i_* statistics was averaged for SNPs in non-overlapping windows of 1 Mb. A total of 2562 informative windows with an average of 16.07 SNPs per window (SD = 3.6) were left after removal of windows with less than five SNPs. We plotted the window-averaged di values across the chromosome, and the top 1% windows of the empirical distribution were defined as putative selection regions. On the other hand, pairwise comparison between BAL and each of the other breeds for XPEHH scores were computed separately with the R package *rehh* [52] using haplotype information. Haplotypes were estimated with *fastphase* 1.4 [53] by using population label information to estimate phased haplotype background and applying the following options for each chromosome: -T10 -C25 -Ku40 -Kl10 -Ki5 -Km1000 -H100, where T is the number of the start of EM algorithm, C is the number of iterations of EM algorithm, Km is the number of SNP loci used for cross-validation, Ki is the interval between values of number of clusters, Ku and Ki are the upper and lower limit of number of clusters, respectively. For comparison of selection signatures and visualization, |XPEHH| scores were transformed into $pXPEHH=-log\left[1-2\left|\phi XPEHH-0.5\right|\right]$ in which ϕ *XPEHH* denotes the Gaussian cumulative distribution function, which was calculated by the pnorm R function. Since the XPEHH scores were approximately normally distributed, *pXPEHH* can be interpreted as log101P. Therefore, *pXPEHH* was the two-sided *p*-value for testing the null hypothesis that no selection occurred [48], and we defined putative selection signals as *pXPEHH* ≥ 5 equivalent to a *p*-value of 0.00001. Common candidate selection regions from all three XPEHH analyses with a minimum of three SNPs were chosen as selection signatures specific to BAL. The selected regions were defined with a maximum base-pair length of approximately 1 Mb.

### 2.5. Gene Annotation and Enrichment Analysis

Genes located within the candidate selection regions for BAL were identified using the UCSC genome browser [54] with the selection of the Oar_v4.0 assembly. Furthermore, functional enrichment analysis was performed on genes identified by both *F_ST_* (*d_i_*) and XPEHH methods separately using the Database for Annotation, Visualization and Integrated Discovery (DAVID) software (https://david.ncifcrf.gov, accessed on 14 February 2022) [55,56]. The gene list was analyzed by selecting the human gene annotations with their background due to low number of recognized genes when sheep gene annotations were selected. The gene ontology annotation category, including biological process (BP) and molecular function (MF) terms, was investigated in this study. The GO TERMS of enriched genes with a *p*-value threshold of ≤0.05 were considered significant, and correction for multiple testing was carried out using the FDR procedure [57]. Overlapping regions of signatures of selection were defined as those regions above the threshold for both methods and within the same chromosomal location [58].

## 3. Results

### 3.1. Population Structure

**Figure 2:**
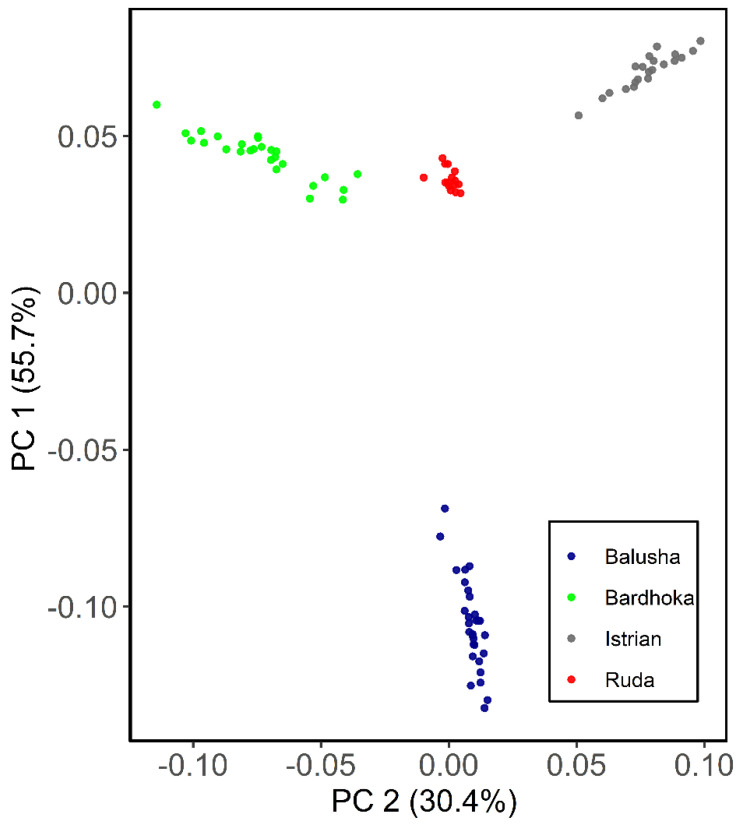
Principal component analysis plot for the first (PC1) and second (PC2) components of Balusha, Bardhoka, Istrian and Ruda sheep breeds. The different breeds are color-coded as mentioned in the legend. Note that the blue Balusha cluster is clearly separated from the rest of the breeds.

**Figure 3:**
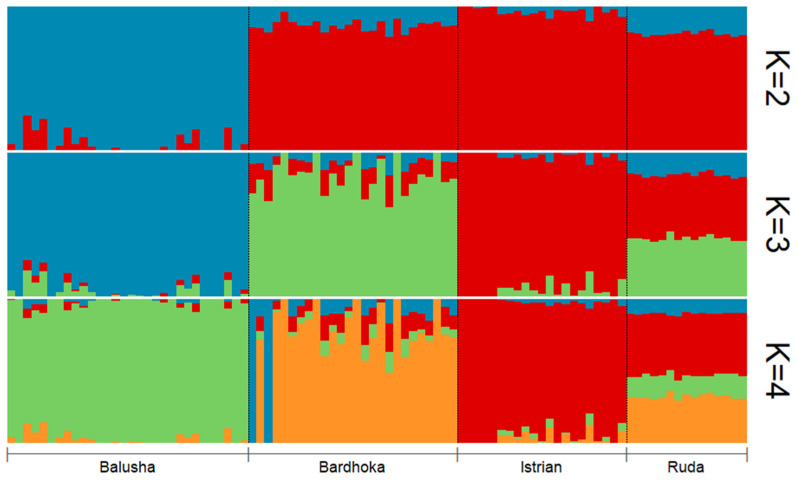
Admixture plot for all sheep breeds (Balusha, Bardhoka, Istrian and Ruda) analyzed in the study. A clear pattern of ancestry is visible for the breeds. Please note that K = 3 was identified as the optimal number of clusters.

Results of genetic diversity indices for the Balkan breeds in this study are given in Table 1. The observed and expected heterozygosity ranged from 0.351 (BAL) to 0.384 (IST) and 0.341 (BAL) to 0.402 (RUD), respectively. Allelic richness was comparable among the sheep breeds with the highest observed in the RUD (1.876) and the lowest in the BAL (1.780). Similarly, the highest private allelic richness was found in RUD (0.012), and the lowest in BAL (0.009). The *F_IS_* for the Balusha (−0.029) and Istrian breeds (−0.009) were observed to be negative with the highest value observed for Ruda (0.054) and the lowest for Istrian. The recent effective population sizes (Ne_5_) were estimated for all breeds and ranged from 25 (BAL) to 35 (BAR), while the effective population sizes 50 generations ago (Ne_50_) varied between 228 in BAL and 324 in BAR. The PCA plot (Figure 2) showed that the first component explained 55.7% of the genetic variability between the breeds. It clearly distinguished the BAL breed from the others. In addition, the second principal component accounted for 30.4% of the variability and clearly separated the other breeds from each other (BAR, IST and RUD). The ADMIXTURE analysis revealed a clear pattern of ancestry for the breeds (Figure 3). Based on cross-validation error, an optimal value of K = 3 clusters was identified. At K = 2, the other breeds were clearly separated from the BAL breed. However, a genetic distinction between the IST breed and the other two breeds (BAR and RUD) was observed at K = 3.

### 3.2. Runs of Homozygosity

**Figure 4:**
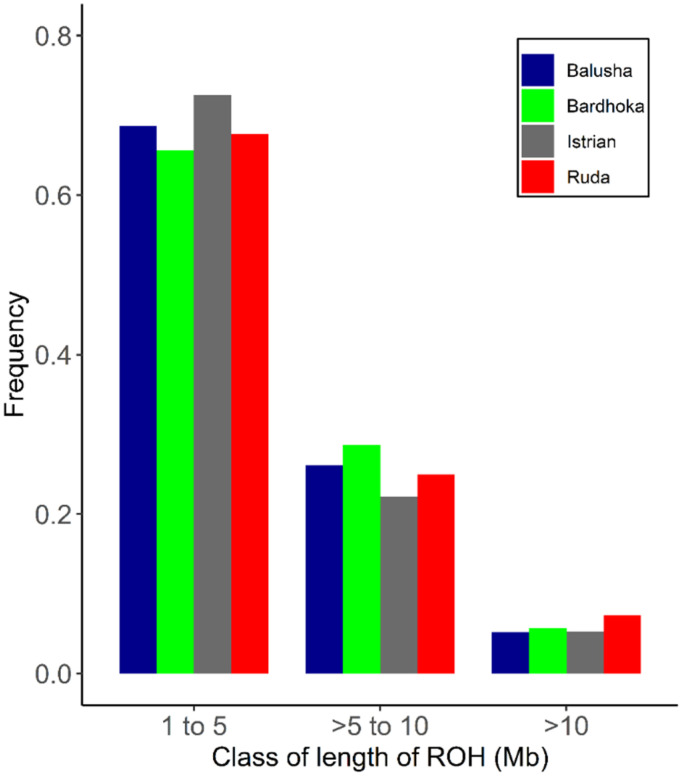
Frequency distribution of number of ROH in varying class lengths and for each breed.

**Figure 5:**
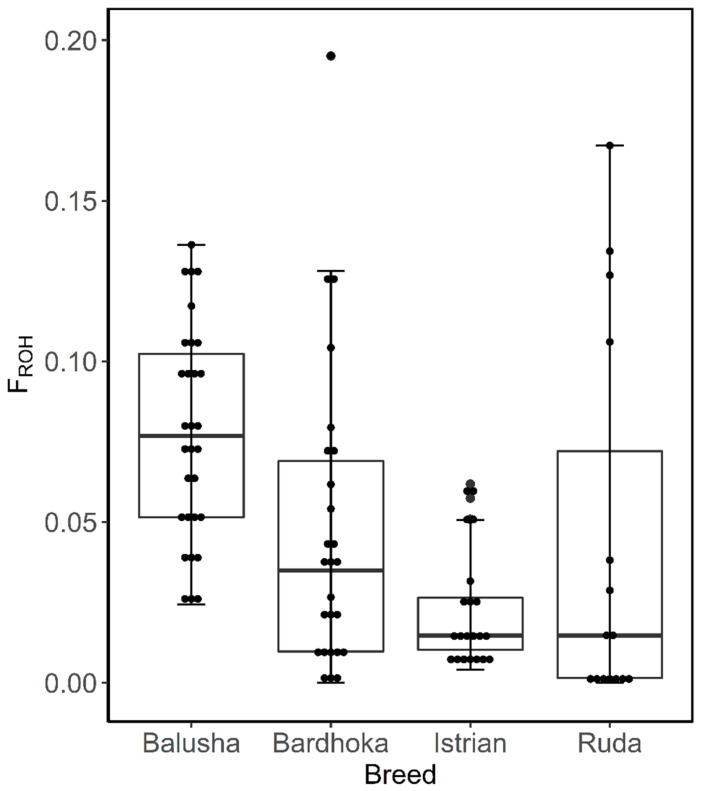
Box plots of within-breed distribution of runs of homozygosity inbreeding coefficient for each sheep breed.

A total of 2562 ROH across all breeds were identified. Out of the 92 animals analyzed, 90 had at least one ROH. The number of ROH varied within and between breeds, with a higher frequency of short ROH segments in all breeds (Figure 4). The mean number of ROH between breeds ranged from 12.67 (IST) to 42.90 (BAL). Likewise, the shortest mean length of ROH was observed in IST (58.05 Mb), and the longest in BAL (204.51 Mb) (Table 2). The frequencies of ROH segments in the different length classes (1–5, >5–10, >10 Mb) were comparable between breeds. The frequencies of ROH in the shortest length class ranged from 65.62% (BAR) to 72.56% (IST), with values of 5.21% (BAL) and 7.35% (RUD) observed in the length class >10 Mb (Figure 5). The mean *F_ROH_* (1 Mb) varied between breeds, with the highest found in the BAL breed (0.077), and the lowest in the IST breed (0.023). The within-breed distribution of *F_ROH_* (1 Mb) is visualized in Figure 5, with the highest median observed in the BAL breed. In addition, mean *F_ROH_* at a minimum ROH length of 5 Mb and 10 Mb were calculated, and the highest *F_ROH_* values of 0.039 (5 Mb) and 0.011 (10 Mb) were found in BAL (Table 2).

### 3.3. Signatures of Selection Based on Locus-Specific Divergence (F_ST_)

**Figure 6:**
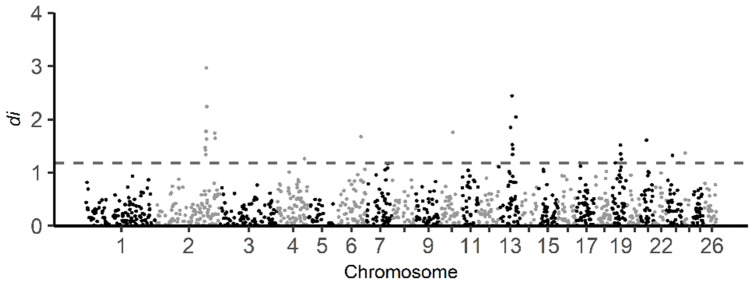
Genome-wide distribution of *d_i_* statistic for all 1 Mb windows across all autosomes. The *d_i_* values calculated from comparison of BAL population against other Balkan breeds (BAR, IST and RUD) in this study. The dashed gray line indicates the top one percent of total informative windows. Note that on chromosomes 2, 4, 6, 10, 13, 19, 21, and 23 signals exceed the threshold.

The *d_i_* statistics were calculated for BAL against all other breeds combined in this study. The distribution of di statistics window values is displayed in Figure 6. A total of 26 informative windows (top 1% of the total number of informative windows) were identified as putative selective sweeps using the di statistics, with significant regions observed on sheep chromosomes 2, 4, 6, 10, 13, 19, 21, 23 and 24 (Table S2a). The informative window with the highest di statistics value (2.97) was found on chromosome 2. Based on the di statistics, 194 genes were identified in the regions under selection (Table S3). Regarding enrichment analysis of genes, 177 of the genes identified from selection signals using the di statistics were recognized by the DAVID software (Table S2b). Furthermore, the result shows that 27 GO TERMS were enriched (*p*-value < 0.05) (Table 3).

### 3.4. Selection of Signatures Based on XPEHH

**Figure 7:**
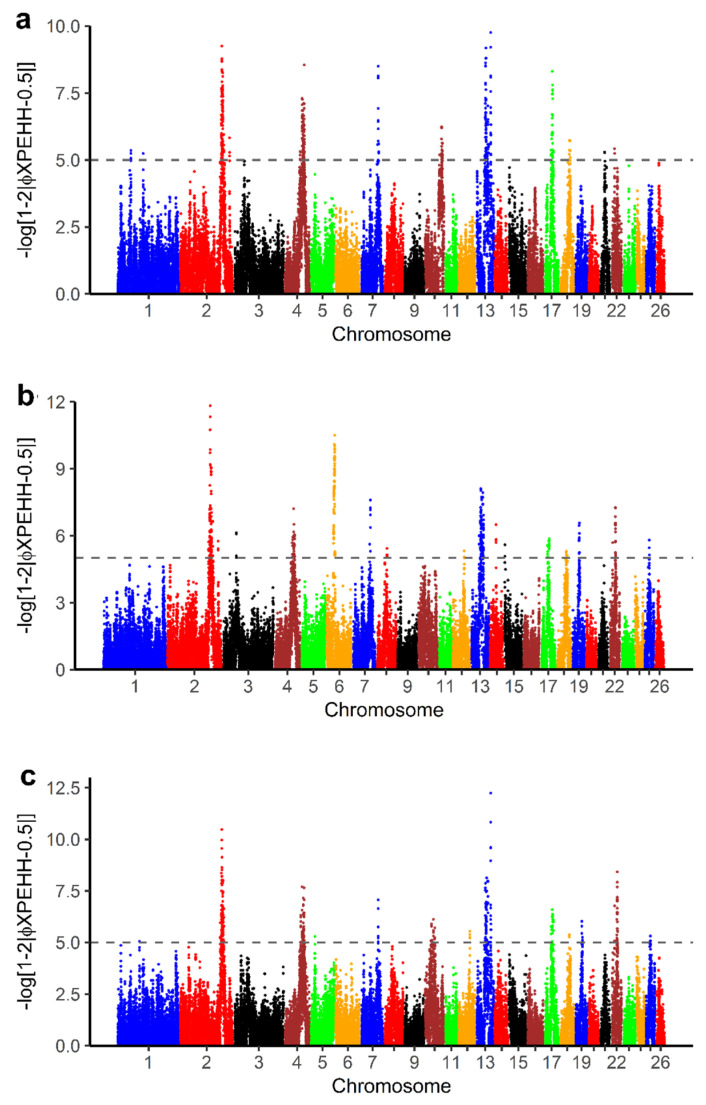
Manhattan plots for cross-population extended haplotype homozygosity (XPEHH) analyses of (**a**) Balusha vs. Bardhoka, (**b**) Balusha vs. Istrian and (**c**) Balusha vs. Ruda. The dashed lines represent the threshold level for significance at *pXPEHH* ≥ 5 equivalent to a *p*-value < 0.00001. Common selection signals were considered as signatures of selection specific for Balusha. Overall they are most obvious in chromosomes 2, 4 and 13.

The *pXPEHH* values for pairwise comparison of BAL with other breeds in this study are displayed in Figure 7. To define selection signals specific to BAL, common significant selection regions with at least three SNPs were selected from the pairwise XPEHH analyses (Table S4a–c). From this selection, we identified 15 candidate selection regions (Table S4d) on OAR 2, 4, 7, 13 and 17. A total of 82 genes were found in selection signals specific to BAL with the XPEHH method (Table S3). Of these, 76 genes were recognized by the DAVID software (Table S4e). The result shows that 13 GO TERMS were significantly enriched (*p*-value < 0.05) (Table 4).

Some overlapping signatures of selections were observed. Regions on sheep chromosomes 2 and 13 were jointly identified by both di statistics and XPEHH. A total of 34 genes were identified in these regions (Table S3).

## 4. Discussion

The Kosovar BAL breed is regarded as an endangered breed [14]. Already, the geographical distribution of the BAL breed is a major negative factor in the risk status of this breed, as the concentration of remaining animals in only two flocks makes them more vulnerable in the unexpected event of, for example, an outbreak of infectious animal disease. Another factor is the small number of breeding individuals remaining and the missing exchange of animals between flocks in concordance with a missing breeding program. Even though farmers try to keep BAL and BAR separate, this proves difficult, especially during the extensive grazing period, which also includes the mating season. This raises difficulties for maintaining the two breeds as purebreds.

The mean expected heterozygosities of the breeds in this study were in concordance with the range reported for other sheep breeds in earlier studies [20,21,22,25]. As expected, the BAL breed had the lowest genetic diversity of the four breeds analyzed in our study. The low He (0.341) observed in BAL is in accordance with the geographical isolation and points to the lack of gene flow between BAL and other breeds in southeastern Europe. This is corroborated by the PCA (Figure 2) and the admixture analysis (Figure 3), clearly differentiating BAL from the other analyzed Balkan breeds. For all breeds analyzed, the effective population sizes at 5 and 50 generations ago were observed to be lower than those reported for sheep breeds from other European or Eurasian regions, e.g., Russian [59] and Kyrgyzstan [20] sheep breeds. The highest effective population size values (Ne_5_ = 35; Ne_50_ = 324) were observed in the BAR breed. This fits very well with a report that this breed is unlike the others widespread across different countries in eastern Europe [60], but still, it points towards the risk of extinction for all analyzed breeds. The low genetic diversity in the BAL breed is in accordance with its low effective population size values (Ne_5_ = 25; Ne_50_ = 228). Moreover, Ne_50_ for the breeds in this study were within the range of ten European and three non-European breeds reported by [21,25]. Furthermore, the mean allelic richness, ranging from 1.780 to 1.876, is slightly lower than values observed in sheep breeds from Russia and Kyrgyzstan [20,59], but within the range observed in worldwide breeds [25]. Even though results from microsatellite analysis are not directly comparable with SNP data, allelic richness and private allelic richness for BAR and IST breeds show similar patterns compared with [33].

Regarding inbreeding, various methods can be employed including *F_IS_* and *F_ROH_* whereby the latter is considered the more precise approach [28]. The BAL population had a negative *F_IS_*, which suggests increased heterozygosity and decreased inbreeding. It is possible that unsupervised crossbreeding with BAR may be responsible. However, the observed *F_ROH_* values and the mean ROH length indicated the presence of inbreeding, which was expected at least for BAL with the highest *F_ROH_* (0.077). The distribution of ROH classes (Figure 4) supports the assumption that mainly historical inbreeding events are relevant for local sheep breeds [21] and only a small proportion of inbreeding (BAL = 14.29%, BAR = 15.22%, IST = 13.04%, RUD = 21.43%) is due to recent events within the last five generations.

The inbreeding status is an important factor in qualifying a breed as endangered, therefore the result for BAL indicates the urgent need to set up a conservation program to reduce inbreeding and increase genetic diversity. The Bela Krajina Pramenka is an example of a local breed in a southeastern European country, Slovenia, for which the setup of a conservation program showed success, enlarging the number of purebred animals from 250 to around 900 within 17 years [61,62].

In BAL, several signatures of selection were detected on different chromosomes, which may result from selection by humans and/or the adaptation to the environment. Chromosomes 2, 13 and 19 showed the strongest signals based on the *F_ST_* (*d_i_*) method, while ovine chromosomes 2, 4 and 13 were prominent based on the XPEHH method. Interestingly, there is no concordance between the genes located in these regions with previously published results of common genes within signatures of selection in ruminants (summarized by [21]). However, four genes on ovine chromosome 2 (*ALS2*, *AOX1*) and 13 (*PPP1R3D*, *SYCP2*) were also found by Manzari et al. [63]. Based on an analysis of Asian and European sheep breeds, several genes with the same chromosomes such as those in our study were identified (Chr. 2: *KIAA2012*, *SUMO1*, Chr. 13: *GNAS*, Chr. 19: *CTNNB1*) [64,65].

**Figure 8:**
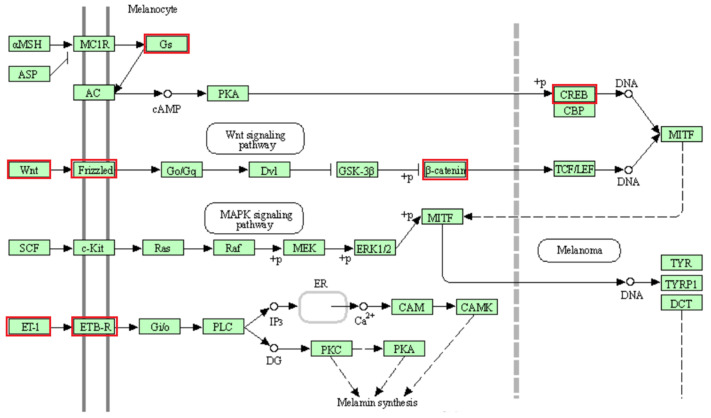
Melanogenesis pathway (KEGG database [76], map04916, truncated to the part relevant for this study). The genes belonging to the proteins highlighted in red were identified in selection signals specific for Balusha. Please note that name of the genes and the corresponding proteins are not always the same (e.g., Frizzled = FZD7, FZD5). All involved proteins are displayed in light green boxes.

GO TERMS analysis identified the groups T cell co-stimulation (GO:0031295) and Wnt signaling pathway (GO:0060071). Potential candidate genes observed within selection signals on chromosome 2 (*WNT4*, *FZD7*, *FZD5*) are implicated in Wnt signaling [66,67], which is an essential process for melanogenesis [68]. Likewise, the *CREB1* gene located within selection signatures on chromosome 2 is involved in the melanin production process by transcriptional activation of the microphthalmia-associated transcription factor (*MITF*) gene [69]. As possible candidate genes in the region of selection signals on chromosome 13, we observed the *GNAS* and *GNAS-AS1* genes, which are involved in the production and regulation, respectively, of G proteins such as the G protein α-subunit (Gs), functioning in melanogenesis as well [70,71,72]. Additionally, within this region of chromosome 13, the endothelin-3 (*EDN3*) gene is located, which is an isoform of the endothelin-1 (*EDN1*) gene [73]. Studies have implicated both genes in the melanogenesis pathway, with equal affinity for endothelin receptor type B (ENDRB) [73,74,75]. EDN3 is also involved in the production of embryonic melanocytes during fetal development [71]. Additionally, the *CTNNB1* (ß-catenin) gene observed in a selection signal region on chromosome 19 is important for the enhancement of TCF/LEF (T cell factor/lymphoid enhancer factor) and subsequent transcriptional activation of the *MITF* gene downstream of the Wnt signaling pathway [67,68]. Our findings reveal that the agouti signaling protein (*ASIP*) and *MITF* genes associated with coat color in animals are located at a distance of 3.1 Mb and 2.2 Mb from the nearest selection signal on chromosome 13 and chromosome 19, respectively. Therefore, due to their participation in the melanogenesis pathway (Figure 8), some or all of the mentioned putative candidate genes on chromosomes 2, 13 and 19 may have an influence on coat color differences between BAL and the other analyzed breeds. This fits well with the predominant difference between BAL and the other breeds, which is the distinct black-colored head of BAL that makes this breed easily recognizable (Figure 1).

**Figure 9:**
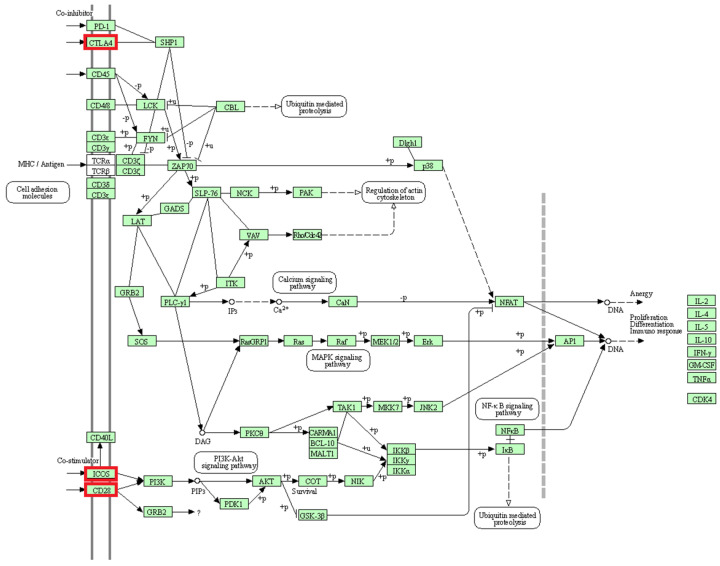
Human T cell receptor signaling pathway (KEGG database [76], hsa04660). The genes belonging to the proteins highlighted in red were identified in selection signals specific for Balusha. All involved proteins are displayed in a light green box.

The activation and survival of T cells are associated with three genes (*CD28*, *CTLA4*, *ICOS*) located in a region with selection signals on chromosome 2 [77] (Figure 9). T cells, as part of the immune system, aid the proper function of immune responses, homeostasis and memory [78,79]. Studies have shown that CD28 provides costimulatory signals, and promotes T cell proliferation, cytokinin production and cell survival [77,80]. Likewise, ICOS is a known co-stimulator of T cell activation, proliferation and differentiation [77,81]. Furthermore, earlier reports revealed that the CTLA4 is a homolog of CD28 and acts as a suppressor of the latter [80,82]. Consequently, increased CTLA4 expression leads to the termination of T cell activation. Interestingly, this study shows that factors of adaptive immunity show differences in signatures of selection between BAL and BAR reared in the same location.

Finally, we identified, as a possible candidate gene, the *CTSZ* gene (located within selection signals on chromosome 13), which was associated with tuberculosis (Mycobacterium tuberculosis) susceptibility in humans [83,84,85]. Investigation of a possible association of this potential candidate gene with paratuberculosis (Mycobacterium avium subspecies paratuberculosis) susceptibility in BAL sheep would be a promising task, as there is an increasing interest in breeding for resistance to infectious diseases in farm animals, including paratuberculosis in ruminants [86]. As it was shown for small ruminant lentiviruses, protective variants against infectious diseases can show a breed-specific distribution e.g., [87]. The findings of this study suggest that attention should be paid to the BAL sheep with the possibility of using them as a source for modern breeding goals such as resistance to infectious diseases, which is a further reason for the conservation of local breeds.

## 5. Conclusions

Reliable knowledge of the diversity between and within breeds is, among others, a requirement for an efficient conservation strategy. Therefore, our study on the genetic diversity of the Kosovar BAL breed provides a basic work for the preservation of this rare local breed and indicates options for its future development. The combination of the results from signatures of selection and the ROH analyses provides a better understanding of how the BAL breed is genetically distinguishable from the other considered sheep breeds originating from the Balkan Peninsula. GO TERMS analysis revealed two interesting groups of genes involved in melanogenesis and T cell co-stimulation, which might interfere with BAL’s unique color pattern and the immune system. The latter could point toward a changed disease susceptibility, which would make BAL a valuable genetic resource for breeding for animal health.

Since our analyses showed that BAL sheep differ strongly from the other compared Pramenka sheep breeds, and the breed is not known to occur in other countries, conservation of the BAL sheep should be given priority over other regional breeds.

## Figures and Tables

**Table 1:** Genetic diversity (LD = r^2^ > 0.5) and effective population size of breeds analyzed in this study.

Breed	*n*	Ho	He	A_R_	pA_R_	*F_IS_*	Ne_5_	Ne_50_
Balusha	30	0.351	0.341	1.780	0.009	−0.029	25	228
Bardhoka	26	0.372	0.378	1.846	0.010	0.018	35	324
Istrian	21	0.384	0.380	1.844	0.011	−0.009	31	277
Ruda	15	0.380	0.402	1.876	0.012	0.054	29	266

**Table 2:** Descriptive statistics for ROH and FROH of breeds analyzed in this study.

	Breed			
	Balusha	Bardhoka	Istrian	Ruda
Number of samples	30	26	21	15
ROH length (Mb)				
Mean (SD)	204.51 (88.30)	124.85 (125.81)	58.05 (48.49)	113.16 (156.06)
Range	64.28–361.00	0–516.50	10.77–163.54	0–442.61
ROH Number				
Mean (S.D)	42.90 (16.31)	25.73 (24.09)	12.67 (9.19)	22.67 (30.35)
Range	15–73	0–101	2–35	0–88
*F_ROH_* (1 Mb)				
Mean	0.077	0.046	0.023	0.042
Range	0.02–0.14	0–0.20	0–0.06	0–0.17
*F_ROH_* (5 Mb)				
Mean	0.039	0.026	0.011	0.024
Range	0–0.08	0–0.12	0–0.03	0–0.10
*F_ROH_* (10 Mb)				
Mean	0.011	0.007	0.003	0.009
Range	0–0.03	0–0.05	0–0.02	0–0.04

**Table 3:** Enriched GO TERMS determined by DAVID from genes identified in selection regions specific to the BAL breed using *F_ST_* (*d_i_*). Please note that GOTERM_MF refers to molecular function and GOTERM_BP refers to biological process.

Category	Term	Genes	*p*-Value	FDR
GOTERM_MF_DIRECT	GO:0016616: oxidoreductase activity, acting on the CH-OH group of donors, NAD or NADP as acceptor	*LDHC, UEVLD, LDHA, MDH1B*	0.000	0.095
GOTERM_BP_DIRECT	GO:0060789: hair follicle placode formation	*CDC42, GNAS, CTNNB1*	0.001	0.425
GOTERM_BP_DIRECT	GO:0030182: neuron differentiation	*EDN3, FZD5, SNPH, FZD7, ID3, WNT4*	0.001	0.425
GOTERM_BP_DIRECT	GO:0007411: axon guidance	*KLF7, NRP2, CREB1, DCC, EPHA8, EPHB2, FEZF2*	0.002	0.425
GOTERM_BP_DIRECT	GO:0060231: mesenchymal to epithelial transition	*TCF15, FZD7, WNT4*	0.002	0.425
GOTERM_MF_DIRECT	GO:0005525: GTP binding	*CDC42, TUBB1, GNAS, GIMAP2, SUCLG2, GIMAP4, GIMAP5, PCK1, GIMAP7, RAB22A*	0.004	0.602
GOTERM_BP_DIRECT	GO:0060070: canonical Wnt signaling pathway	*CDC42, FZD5, FZD7, CTNNB1, WNT4*	0.004	0.769
GOTERM_BP_DIRECT	GO:0019752: carboxylic acid metabolic process	*LDHC, UEVLD, LDHA*	0.005	0.769
GOTERM_BP_DIRECT	GO:0006366: transcription from RNA polymerase II promoter	*BMPR2, CREB1, GTF2H1, MYBL2, ZNF831, NELFCD, SOX12, FEZF2, POU4F1, CARF, CTCFL*	0.007	1.000
GOTERM_BP_DIRECT	GO:0045669: positive regulation of osteoblast differentiation	*BMPR2, GNAS, CTNNB1, WNT4*	0.012	1.000
GOTERM_BP_DIRECT	GO:0072033: renal vesicle formation	*CTNNB1, WNT4*	0.016	1.000
GOTERM_BP_DIRECT	GO:0048145: regulation of fibroblast proliferation	*CREB1, CTNNB1*	0.016	1.000
GOTERM_BP_DIRECT	GO:0051726: regulation of cell cycle	*L3MBTL1, ID3, E2F2, MYBL2, CDK15*	0.017	1.000

**Table 4:** Enriched GO TERMS determined by DAVID from genes identified in selection regions specific to the BAL breed using XPEHH. Please note that GOTERM_MF refers to molecular function and GOTERM_BP refers to biological process.

Category	Term	Genes	*p* Value	FDR
GOTERM_MF_DIRECT	GO:0005212: structural constituent of eye lens	*CRYGC, CRYGD, CRYGA, CRYGB*	0.000	0.005
GOTERM_MF_DIRECT	GO:0004181: metallocarboxypeptidase activity	*CPA2, CPA1, CPO, CPA5*	0.000	0.005
GOTERM_MF_DIRECT	GO:0004415: hyalurononglucosaminidase activity	*HYAL4, SPAM1*	0.023	0.666
GOTERM_MF_DIRECT	GO:0016790: thiolester hydrolase activity	*ACOT2, ACOT1*	0.023	0.666
GOTERM_BP_DIRECT	GO:2000601: positive regulation of Arp2/3 complex-mediated actin nucleation	*ABI2, WASL*	0.024	1.000
GOTERM_BP_DIRECT	GO:0007015: actin filament organization	*FSCN3, WASL, LMOD2*	0.026	1.000
GOTERM_BP_DIRECT	GO:0006508: proteolysis	*CPA2, CPA1, CPO, CTSZ, ADAM23, CPA5*	0.029	1.000
GOTERM_BP_DIRECT	GO:0031295: T cell costimulation	*CD28, CTLA4, ICOS*	0.030	1.000
GOTERM_BP_DIRECT	GO:0007601: visual perception	*CRYGC, CRYGD, CRYGA, CRYGB*	0.032	1.000
GOTERM_MF_DIRECT	GO:0016290: palmitoyl-CoA hydrolase activity	*ACOT2, ACOT1*	0.035	0.832
GOTERM_BP_DIRECT	GO:0008154: actin polymerization or depolymerization	*ABI2, WASL*	0.041	1.000
GOTERM_BP_DIRECT	GO:0000038: very long-chain fatty acid metabolic process	*ACOT2, ACOT1*	0.047	1.000
GOTERM_MF_DIRECT	GO:0047617: acyl-CoA hydrolase activity	*ACOT2, ACOT1*	0.048	0.909

## Data Availability

Data supporting reported results are provided in Supplementary Tables S1–S3.

## Supplementary Materials

The following supporting information can be downloaded at: https://www.mdpi.com/article/10.3390/genes13050866/s1, Table S1: Information on sample numbers and SNP data. Table S2: (a–b) Significant regions of selection from *F_ST_* analysis. Table S3: Annotated genes identified in selection signatures specific to the BAL breed detected by *F_ST_* (*d_i_*) and XPEHH. Table S4: (a–e) Significant regions of selection from XPEHH analysis.
